# Supplementary material for: Comparative genomics provides new insights into the diversity, physiology, and sexuality of the only industrially exploited tremellomycete: Phaffia rhodozyma
Source: BMC Genomics. 2016 Nov 9;17:901. doi: 10.1186/s12864-016-3244-7 (PMC5103461; doi:10.1186/s12864-016-3244-7)
Supplement: Additional file 6: — List of orphan genes with links to PFAM (related to Additional file 1: Table S1). (ZIP 1428 kb) [file 12864_2016_3244_MOESM6_ESM.zip › BLAST_HTML_FTR/G03335_P.html]

BLAST Search Results


```
BLASTP 2.2.27+


Reference:
Stephen F. Altschul, Thomas L. Madden, Alejandro A. Schäffer,
Jinghui Zhang, Zheng Zhang, Webb Miller, and David J. Lipman (1997),
"Gapped BLAST and PSI-BLAST: a new generation of protein database
search programs", Nucleic Acids Res. 25:3389-3402.


Reference for
composition-based statistics:
Alejandro A. Schäffer, L. Aravind, Thomas L. Madden, Sergei
Shavirin, John L. Spouge, Yuri I. Wolf, Eugene V. Koonin, and
Stephen F. Altschul (2001), "Improving the accuracy of PSI-BLAST
protein database searches with composition-based statistics and
other refinements", Nucleic Acids Res. 29:2994-3005.


Database: nr
           71,551,133 sequences; 26,053,659,533 total letters


Query= G03335_P

Length=117
                                                                      Score     E
Sequences producing significant alignments:                          (Bits)  Value

emb|CED85203.1|  bluf domain protein [Xanthophyllomyces dendrorhous]   146    7e-41
emb|CED83127.1|  bluf domain protein [Xanthophyllomyces dendrorhous]  69.7    5e-12
ref|XP_007876148.1|  hypothetical protein PFL1_00463 [Pseudozyma ...  37.7    1.3  
gb|EST09814.1|  hypothetical protein PSEUBRA_SCAF1g00238 [Pseudoz...  35.8    6.1  


 >emb|CED85203.1| bluf domain protein [Xanthophyllomyces dendrorhous]
Length=230

 Score =  146 bits (369),  Expect = 7e-41, Method: Compositional matrix adjust.
 Identities = 105/109 (96%), Positives = 106/109 (97%), Gaps = 0/109 (0%)

Query  1    MSRADSSPTPSSASSSSSSSASSSTSTYNVNASSSSRDNHLATARTECLPVPAGEQNNDD  60
            MSRADSSPTPSSASSSSSSSASSSTSTYNVNASSSSRDNHLATARTECLPVPAGEQNNDD
Sbjct  1    MSRADSSPTPSSASSSSSSSASSSTSTYNVNASSSSRDNHLATARTECLPVPAGEQNNDD  60

Query  61   DSEVDESDIFQLVYCSSSISPAMPRDDLIDILSVCRRNNAAAAIISSIM  109
            DSEVDESDIFQLVYCSSSISPAMPRDDLIDILSVCRRNNAAAAI   +M
Sbjct  61   DSEVDESDIFQLVYCSSSISPAMPRDDLIDILSVCRRNNAAAAISGLLM  109


>emb|CED83127.1| bluf domain protein [Xanthophyllomyces dendrorhous]
Length=232

 Score = 69.7 bits (169),  Expect = 5e-12, Method: Compositional matrix adjust.
 Identities = 34/59 (58%), Positives = 43/59 (73%), Gaps = 1/59 (2%)

Query  52   PAGEQNNDDD-SEVDESDIFQLVYCSSSISPAMPRDDLIDILSVCRRNNAAAAIISSIM  109
            P GE +  +   EVDESDIFQLV+CSSS+SPA+ + D+ DI+ VCRRNNA  AI   +M
Sbjct  53   PNGEIDEHEPLGEVDESDIFQLVFCSSSVSPALSKADMADIIEVCRRNNAIDAISGLLM  111


>ref|XP_007876148.1| hypothetical protein PFL1_00463 [Pseudozyma flocculosa PF-1]
 gb|EPQ32266.1| hypothetical protein PFL1_00463 [Pseudozyma flocculosa PF-1]
Length=297

 Score = 37.7 bits (86),  Expect = 1.3, Method: Compositional matrix adjust.
 Identities = 15/41 (37%), Positives = 26/41 (63%), Gaps = 0/41 (0%)

Query  69   IFQLVYCSSSISPAMPRDDLIDILSVCRRNNAAAAIISSIM  109
            + Q+VY SS+++  + R+++ DIL   RRNNA   I   ++
Sbjct  97   VLQVVYTSSALTRHVSREEISDILQHSRRNNARRGITGLLL  137


>gb|EST09814.1| hypothetical protein PSEUBRA_SCAF1g00238 [Pseudozyma brasiliensis 
GHG001]
Length=299

 Score = 35.8 bits (81),  Expect = 6.1, Method: Compositional matrix adjust.
 Identities = 17/42 (40%), Positives = 24/42 (57%), Gaps = 0/42 (0%)

Query  68   DIFQLVYCSSSISPAMPRDDLIDILSVCRRNNAAAAIISSIM  109
            D+ Q+VY SS+ S  MP D++  IL   R NN A  I   ++
Sbjct  121  DLIQVVYTSSAKSRHMPLDEVKQILRASRSNNTALGITGLLL  162


Lambda      K        H        a         alpha
   0.311    0.120    0.324    0.792     4.96 

Gapped
Lambda      K        H        a         alpha    sigma
   0.267   0.0410    0.140     1.90     42.6     43.6 

Effective search space used: 639098023296


  Database: nr
    Posted date:  Sep 23, 2015 12:05 AM
  Number of letters in database: 26,053,659,533
  Number of sequences in database:  71,551,133


Matrix: BLOSUM62
Gap Penalties: Existence: 11, Extension: 1
Neighboring words threshold: 11
Window for multiple hits: 40
```
